# Supplementary material for: Fatigue and Cognitive Dysfunction Are Associated with Occupational Status in Post-COVID Syndrome
Source: Int J Environ Res Public Health. 2022 Oct 16;19(20):13368. doi: 10.3390/ijerph192013368 (PMC9603617; doi:10.3390/ijerph192013368)
Supplement: Supplementary file 1 [file ijerph-19-13368-s001.zip › ijerph-1937114-supplementary.pdf]

File S1. SURVEY FOR WORK ASSESSMENT

1) Indicate your level of education:

a. No education
b. Primary education
c. Secondary education
d. University education (Bachelor/Master/Doctorate)

2) Indicate your employment status before diagnosis of the disease?

a. Active
   Specify your work profession: ____________________________.
b. Unemployed
   Specify what has been your working profession: _____________________.
c. Student
d. Retired
e. Disability

3) Indicate what is, at currently, your main professional activity: ___________________.

ESCO classification (European Skills, Competences, Qualifications and Occupations) to categorize professional categories (ESCO, 2017):

- Group 0: "Armed forces occupations"
- Group 1: "Managers professionals"
- Group 2: "Technicians and associate professionals"
- Group 3: "Clerical support workers"
- Group 4: "Service and sales workers"
- Group 5: "Skilled agricultural"
- Group 6: "Forestry and fishery workers"
- Group 7: "Craft and related trades workers"
- Group 8: "Plant and machine operators and assemblers"
- Group 9: "Elementary occupations"

4) Have you needed to request sick leave due to symptoms of the disease?

a. Yes
b. No

If the answer to the previous question was *"yes"*, please specify how long you have been or have been on sick leave (INDICATE MONTHS). Otherwise, do not answer.

___________________.

5) Do the symptoms of the disease interfere with your professional career?

a. Yes
b. No

If the answer to the previous question was *"yes"*, please indicate below what were those symptoms. If not, do not answer?

- Fatigue
- Weakness
- Sleep disorders
- Headache
- Dizziness

- Dyspnea
- Attention and concentration /memory problems o Cognitive complaints
- Language problems
- Anxiety/depression
- Others:

Specify if there is any other symptom affecting your work performance that was not mentioned in the previous question. If not, do not answer.

6) What would you say is the most disabling symptom in your professional career? (ONLY INDICATE ONE SYMPTOM)

ANSWER THE FOLLOWING QUESTIONS ONLY IF YOU ARE STILL IN EMPLOYMENT:

1) Have you had to reduce your working hours due to the symptoms of the disease?
   a. Yes
   b. No

2) Only if you answered yes to the previous question, please specify the type of employment you currently have. Otherwise, do not answer.
   - Works full time, but in a less demanding position.
   - Works part-time.
   - Works between 25% and 50%.
   - Works less than 25%.

3) Have you needed to make any accommodations in your job?
   a. Yes
   b. No

4) If you answered "yes" to the previous question, please specify which adaptations have been made. Otherwise, do not answer.
- More breaks
- Telework
- Cognitive aids
- Position change

(the need to make adaptations but did not have that possibility)

Supplementary Table S1. Demographic and occupational outcomes according to the three most frequent ISCO groups

	ISCO Group 2	*ISCO Group 3*	*ISCO Group 5*
Number of patients	44	15	10
Age	45.20±8.40	50.40±6.06	45.50±8.22
Sex (women)	38 (86.4%)	14 (93.3%)	9 (90%)
Education (years)	17.80±1.00	14.27±3.17	14.30±2.98
Time since onset (months)	21.34±6.14	20.40±6.76	21.2±7.00
Required sick leave	37 (84.1%)	13 (86.7%)	9 (90%)
Not returned to work	25 (56.8%)	7 (46.7%)	4 (40%)
Most disabling symptom	Cognition (47.7%), fatigue (31.8%)	Cognition (60%), Fatigue (20%)	Cognition (30%), Fatigue (30%), Dyspnea (20%)

Supplementary Table S2. Comparison of patients on active working or not at the moment of the assessment (excluding those patients that required ventilatory assistance or ICU admission).

	Returned to work (n=38)	Not returned to work (n=35)	p-value
	Demographic factors		
Age	45.68±8.58	46.37±7.48	0.732
Sex (women)	34 (89.5%)	31 (88.6%)	0.597
Education (years)	15.66±3.14	16.46±3.01	0.192
Time since onset	21.84±6.61	20.05±6.45	0.167
	Risk factors and clinical characteristics of acute disease		
Arterial hypertension	6 (15.8%)	6 (17.1%)	0.876
Diabetes mellitus	4 (10.5%)	1 (2.9%)	0.359
Dyslipidemia	10 (26.3%)	7 (20.0%)	0.524
Hospital admission	5 (13.2%)	6 (17.1%)	0.634
	Fatigue and neuropsychiatric scales		
MFIS (total)	55.24±15.19	67.57±10.11	**<0.001**
MFIS (physical)	25.24±7.04	30.46±4.99	**<0.001**
MFIS (cognitive)	24.68±8.90	30.49±5.36	**0.004**
MFIS (psychosocial)	5.00±2.29	6.43±1.72	**0.004**
BDI	14.65±8.67	16.80±7.03	0.097
STAI-State	40.05±9.54	41.77±10.48	0.554
STAI-Trait	47.07±12.01	49.91±12.29	0.371
PSQI	10.61±3.94	12.17±4.33	0.113
BSIT	9.53±2.24	9.59±1.65	0.801
	Cognitive testing		
Digit span forward	5.79±1.37	5.66±1.34	0.560
Digit span backward	4.05±1.22	4.34±1.21	0.270
Corsi forward	5.87±1.09	5.57±1.09	0.269
Corsi backward	5.11±1.26	4.89±0.93	0.433
SDMT	44.42±13.73	39.54±14.93	0.122
Boston Naming Test	53.42±4.62	53.37±4.57	0.938
ROCF copy(accuracy)	33.92±2.83	34.38±1.75	0.670
ROCF copy (time)	132.63±65.33	122.11±42.53	0.420
ROCF 3 min	20.03±6.11	22.57±6.34	0.084
ROCF 30 min	19.90±6.04	21.02±6.14	0.353
ROCF recognition	19.16±2.47	19.51±3.45	0.794
Stroop W	95.50±22.58	85.09±25.63	0.103
Stroop C	66.84±15.76	58.20±16.85	**0.046**
Stroop W-C	42.00±13.42	33.94±11.31	**0.014**
FCSRT (Free recall Trial 1)	7.97±2.08	8.09±2.31	0.754
FCSRT (Total free recall)	28.37±7.46	28.09±6.28	0.744
FCSRT (Total recall)	42.18±7.18	41.46±5.24	0.185
FCSRT (Delayed free recall)	10.29±3.01	10.11±3.24	0.100
FCSRT (Delayed total recall)	14.50±2.68	14.17±2.10	0.196
Verbal fluency Animals	22.08±6.39	22.26±6.78	0.723

Verbal fluency "P"	15.84±4.32	17.31±5.05	0.217
VOSP Object decision	16.82±2.27	16.69±2.02	0.603
VOSP Progressive silhouettes	8.39±2.95	8.17±2.07	0.942
VOSP Position discrimination	19.29±1.91	19.20±1.93	0.783
VOSP Number location	9.00±1.69	9.00±1.30	0.824
JLO	23.45±5.59	23.34±4.53	0.872

MFIS = Modified Fatigue Impact Scale; BDI = Beck Depression Inventory-II; STAI= State-Trait Anxiety Inventory; PSQI= Pittsburgh Sleep Quality Index; BSIT= Brief Smell Identification Test; SDMT= Symbol Digit Modality Test; ROCF = Rey-Osterrieth Complex Figure; Stroop W= Stroop Words; Stroop C = Stroop Color; Stroop W-C= Stroop Word-Color; FCSRT= Free and Cued Selective Reminding Test; VOSP = Visual Object and Space Perception Battery; JLO= Judgement Line Orientation test. Statistically significant p-values are shown in **bold**.

Supplementary Table S3. Comparison between patients that required sick leave or not.

	Required sick leave (n=63)	Did not require sick leave (n=14)	U/χ²	p
		Demographic factors		
Age	46.52±8.02	45.36±7.94	U= 410.50	0.687
Sex (women)	54 (85.7%)	13 (92.9%)	χ^2= 0.517	0.679
Education (years)	16.22±3.013	15.79±3.23	U=408.50	0.600
Time since onset (months)	21.49±6.05	17.21±7.51	U=271.50	0.025
		Clinical characteristics of acute disease		
Arterial hypertension	11 (17.5%)	1 (7.1%)	χ^2= 0.92	0.684
Diabetes mellitus	5 (7.9%)	1 (7.1%)	χ^2= 0.01	1.000
Dyslipidemia	18 (28.6%)	1 (7.1%)	χ^2= 2.83	0.168
Hospital admission	14 (22.2%)	1 (7.1%)	χ^2= 1.66	0.28
Ventilatory assistance	4 (6.3%)	0 (0%)	χ^2= 0.93	1.00
ICU admission	3 (4.8%)	0 (0%)	χ^2= 0.69	1.00
		Fatigue and neuropsychiatric scales		
MFIS (total)	63.59±13.16	51.50±14.36	U=228.00	**<0.001**
MFIS (physical)	28.57±6.30	24.29±637	U=253.50	**0.013**
MFIS (cognitive)	28.60±7.64	23.43±7.63	U=263.50	**0.019**
MFIS (psychosocial)	6.11±1.82	3.79±2.35	U=192.50	**0.001**
BDI	16.71±7.99	12.50±7.49	U=279.50	**0.038**
STAI-State	41.94±10.73	38.71±8.10	U=382.50	0.439
STAI-Trait	49.43±11.96	44.57±13.39	U=335.00	0.161
PSQI	11.56±4.28	10.50±3.61	U=334.50	0.158
BSIT	9.66±2.04	8.69±2.92	U=319.00	0.261
		Cognitive testing		
Digit span forward	5.79±1.53	5.29±0.825	U=334.50	0.150
Digit span backward	4.17±1.289	4.36±1.082	U=388.50	0.473
Corsi forward	5.59±1.200	6.29±0.994	U=296.00	**0.047**
Corsi backward	4.84±1.139	5.64±1.008	U=252.50	**0.010**
SDMT	40.63±14.48	48.43±12.64	U=306.50	0.075
Boston Naming Test	53.75±4.50	52.57±4.86	U=376.50	0.393
ROCF copy (accuracy)	34.07±1.95	34.07±4.02	U=336.00	0.149
ROCF copy (time)	127.59±56.71	127.00±47.63	U=427.50	0.858
ROCF 3 min	20.77±6.28	22.21±6.50	U=369.00	0.341
ROCF 30 min	19.78±5.67	22.60±7.35	U=341.50	0.188
ROCF recognition	19.27±2.81	19.64±3.31	U=352.50	0.238
Stroop W	86.60±25.64	101.57±18.47	U=295.00	0.054
Stroop C	60.08±17.50	71.57±9.70	U=248.50	**0.011**
Stroop W-C	36.84±13.66	41.86±9.90	U=312.00	0.088
FCSRT (Free recall Trial 1)	7.59±1.90	9.64±2.53	U=224.50	**0.004**
FCSRT (Total free recall)	27.51±6.86	31.00±5.81	U=315.50	0.097
FCSRT (Total recall)	41.29±6.55	44.14±4.84	U=290.00	**0.045**
FCSRT (Delayed free recall)	10.14±3.07	10.36±2.89	U=420.00	0.780

FCSRT (Delayed total recall)	14.22±2.44	1493±1.94	U=357.00	0.240
Verbal fluency Animals	21.46±6.66	24.07±5.28	U=315.00	0.095
Verbal fluency P	16.33±4.87	17.14±3.88	U=392.50	0.521
VOSP Object decision	16.70±2.24	16.79±1.67	U=437.00	0.957
VOSP Progressive silhouettes	8.33±2.50	8.00±2.51	U=427.50	0.857
VOSP Position discrimination	19.10±2.05	19.93±0.26	U=327.50	0.055
VOSP Number location	8.94±1.59	9.29±0.91	U=391.50	0.487
JLO	23.17±4.52	24.57±7.34	U=399.00	0.578

MFIS = Modified Fatigue Impact Scale; BDI = Beck Depression Inventory-II; STAI= State-Trait Anxiety Inventory; PSQI= Pittsburgh Sleep Quality Index; BSIT= Brief Smell Identification Test; SDMT= Symbol Digit Modality Test; ROCF = Rey-Osterrieth Complex Figure; Stroop W= Stroop Words; Stroop C = Stroop Color; Stroop W-C= Stroop Word-Color; FCSRT= Free and Cued Selective Reminding Test; VOSP = Visual Object and Space Perception Battery; JLO= Judgement Line Orientation test. Statistically significant p-values are shown in **bold**.

Supplementary Table S4. Comparison between patients that required sick leave or not (excluding those patients that required ventilatory assistance or ICU admission)

	Required sick leave (n=59)	Did not require sick leave (n=14)	p-value
	Demographic factors		
Age	46.17±8.10	45.36±7.94	0.828
Sex (women)	52 (88.1%)	13 (92.9%)	0.520
Education (years)	16.10±3.07	15.79±3.23	0.705
Time since onset (months)	21.88±6.03	17.21±7.51	**0.014**
	Clinical characteristics of acute disease		
Arterial hypertension	11 (18.6%)	1 (7.1%)	0.440
Diabetes mellitus	4 (6.8%)	1 (7.1%)	1.000
Dyslipidemia	16 (27.1%)	1 (7.1%)	0.165
Hospital admission	10 (16.9%)	1 (7.1%)	0.679
	Fatigue and neuropsychiatric scales		
MFIS (total)	63.44±13.45	51.50±14.36	**0.006**
MFIS (physical)	28.56±6.49	24.29±637	**0.012**
MFIS (cognitive)	28.42±7.75	23.43±7.63	**0.025**
MFIS (psychosocial)	6.14±1.84	3.79±2.35	**0.001**
BDI	16.47±7.87	12.50±7.49	**0.048**
STAI-State	41.39±10.36	38.71±8.10	0.523
STAI-Trait	49.36±11.76	44.57±13.39	0.165
PSQI	11.56±4.31	10.50±3.61	0.158
BSIT	9.75±1.65	8.69±2.92	0.268
	Cognitive testing		
Digit span forward	5.83±1.44	5.29±0.825	0.116
Digit span backward	4.15±1.25	4.36±1.082	0.414
Corsi forward	5.59±1.08	6.29±0.994	**0.038**
Corsi backward	4.85±1.09	5.64±1.008	**0.009**
SDMT	40.58±14.51	48.43±12.64	0.069
Boston Naming Test	53.59±4.51	52.57±4.86	0.486
ROCF copy (accuracy)	34.16±1.83	34.07±4.02	0.172
ROCF copy (time)	127.73±57.52	127.00±47.63	0.839
ROCF 3 min	21.02±6.30	22.21±6.50	0.424
ROCF 30 min	19.93±5.68	22.60±7.35	0.228
ROCF recognition	19.25±2.90	19.64±3.31	0.244
Stroop W	87.88±25.12	101.57±18.47	0.072
Stroop C	60.59±17.43	71.57±9.70	**0.015**
Stroop W-C	37.25±13.57	41.86±9.90	0.105
FCSRT (Free recall Trial 1)	7.64±1.92	9.64±2.53	**0.005**
FCSRT (Total free recall)	27.58±6.99	31.00±5.81	0.116
FCSRT (Total recall)	41.29±6.50	44.14±4.84	**0.046**
FCSRT (Delayed free recall)	10.17±3.17	10.36±2.89	0.849
FCSRT (Delayed total recall)	14.20±2.51	1493±1.94	0.270

Verbal fluency Animals	21.71±6.76	24.07±5.28	0.129
Verbal fluency P	16.41±4.90	17.14±3.88	0.560
VOSP Object decision	16.75±2.25	16.79±1.67	0.898
VOSP Progressive silhouettes	8.36±2.58	8.00±2.51	0.832
VOSP Position discrimination	19.08±2.09	19.93±0.26	0.053
VOSP Number location	8.93±1.61	9.29±0.91	0.480
JLO	23.12±4.41	24.57±7.34	0.555

MFIS = Modified Fatigue Impact Scale; BDI = Beck Depression Inventory-II; STAI= State-Trait Anxiety Inventory; PSQI= Pittsburgh Sleep Quality Index; BSIT= Brief Smell Identification Test; SDMT= Symbol Digit Modality Test; ROCF = Rey-Osterrieth Complex Figure; Stroop W= Stroop Words; Stroop C = Stroop Color; Stroop W-C= Stroop Word-Color; FCSRT= Free and Cued Selective Reminding Test; VOSP = Visual Object and Space Perception Battery; JLO= Judgement Line Orientation test. Statistically significant p-values are shown in **bold**.
